# TB preventive treatment coverage among children under six years of age in TB-affected households

**DOI:** 10.5588/ijtldopen.25.0207

**Published:** 2025-11-12

**Authors:** A. Kadam, A.H. Moe, M.S. Barthwal, G. Lele, A. Kakrani, V. Mave, N. Suryavanshi, J.E. Golub, S.R. Cox, on behelf of the TB Aftermath study team, A. Mahajan, Y. Bhosale, S. Dugga, P. Kadam, S. Jadhav, V. Bodhe, P. Bhalchim, S. Rathod, V. Dhamale, N. Sonawane, P. Ambekar, A. Bhalchim, A. Patole

**Affiliations:** 1 Johns Hopkins Center for Infectious Diseases in India (CIDI), Pune, India;; 2 Johns Hopkins School of Medicine, Division of Infectious Diseases, Baltimore, USA;; 3 Dr. D.Y. Patil Medical College, Hospital and Research Centre, Pimpri, Pune, India;; 4 Johns Hopkins Bloomberg School of Public Health, International Health, Baltimore, USA.

**Keywords:** tuberculosis, India, TB infection, household contacts, post-treatment

Dear Editor,

An estimated one-quarter of the global population is infected with TB and each year, over 10 million people develop TB disease (TBD).^1,2^ India has the highest TB burden globally with 40% of its population estimated to be infected,^3^ and around 2.8 million people developing TBD in 2023.^2^ Individuals with TB infection (TBI) face a 5–10% lifetime risk of progressing to TBD and are at highest risk of becoming ill within two years of exposure.^4–6^ Young children in close contact with people with TB (PWTB) are at particularly high risk of rapid progression to TBD.^5,7^ TB preventive treatment (TPT) can safely and effectively protect child contacts of PWTB from TBD.^8^ India’s National Strategic Plan for TB Elimination (2017–2025) prioritizes child contacts under six for TB screening and TPT provision, with a target of 95% coverage.^9^ While TPT coverage in this group has been steadily rising in recent years, it has not exceeded 60%^10^ and there is limited data available on factors influencing uptake among child contacts in India. In 2022, India’s National TB Elimination Program (NTEP) introduced new guidance extending the TPT recommendation to all the household contacts of pulmonary TB (PTB) patients with continued emphasis on children <6 years. Additionally, to increase uptake, guidance states that contacts missed during initial screening may be re-screened and offered TPT during the index patient’s post-treatment follow-up period (i.e., approximately 2 years after exposure).^11^ The recommended regimens are 6 months of daily isoniazid (6H) for all contacts or 3 months of weekly isoniazid and rifapentine (3HP) for contacts older than two years. Given the prioritization of children under six for TPT and the expanded scope of the new guidance, we sought to characterize TPT coverage of children under six in TB-affected households during post-treatment longitudinal follow-up and to identify the factors influencing TPT coverage in this group.

This study is nested within the TBAftermath trial, which compares the effectiveness, cost-effectiveness, and feasibility of phone versus home-based TB screening in the post-treatment period in Pune, Maharashtra, India.^12^

From January 2021 to September 2023, the TB Aftermath study consented and enrolled 1,076 TB survivors (≥18 years) within 60 days of completing treatment, with follow-up every six months for 18 months for both arms. At enrollment, we asked the TB survivor about TPT coverage among child contacts under six during their recent treatment course and about the reasons for non-initiation of TPT. We referred potentially eligible children who had not yet received TPT to the NTEP for further evaluation, and communicated this referral to the TB survivors (and accompanying adult household member, if any). During post-treatment follow-up, household contacts of all ages were enrolled and their sociodemographic data were collected after obtaining informed consent from adults and from parents or caregivers of children. At the final 18-month home visit for participants in both arms, we reassessed TPT coverage among these children and reasons for non-initiation of TPT based on reports from TB survivors. As needed, our study staff validated these data with the primary caregiver if available. For children who did initiate TPT, collecting adherence/completion data was outside the scope of our study. For the primary analysis, we compared the characteristics of children who received TPT with those who had not received TPT during the TB survivor’s recent treatment course. We used the Wilcoxon rank-sum test for continuous variables and Pearson’s chi-squared test or Fisher’s exact test for categorical variables, depending on sample size. We categorized the self-reported reasons (collected using structured questionnaire) for non-initiation of TPT as being provider-related or family-related, and used Pearson’s chi-squared test to compare these reasons by family type, sex, and residence. For our secondary analysis, we assessed longitudinal changes in TPT coverage, after referral, at 18-month visit. We also reassessed the reasons for non-initiation of TPT post-treatment compared to reasons for non-initiation during the TB survivor’s recent treatment course. We defined statistical significance as a p-value less than 0.05.

At enrollment, 267 (25%) TB survivors were living with household contacts under six years old during their recent treatment, and 154 (58%) had at least one child who received TPT. Among the 338 children under six who were enumerated, four (1%) were diagnosed with active TB during the TB survivor’s recent treatment and thus, were ineligible for TPT. Among the remaining 334 children, the median age was 3 years (IQR, 2–5), 163 (49%) were female, 240 (72%) had a parent who was a TB survivor, 152 (46%) lived with a nuclear family, 215 (64%) lived in urban areas, and 221 (66%) were exposed to PTB. Of 334 children, 191 (57%) received TPT, the majority of whom had been exposed to PTB (148, 77%). Of 221 exposed to PTB, 207 were exposed to microbiologically-confirmed PTB, and of these, 139 (67%) received TPT. Though not recommended in the guidelines, 43 (38%) of 113 child contacts exposed to extrapulmonary TB (EPTB) received TPT. Compared to older children (≥1 year of age), a significantly higher proportion of children under one year did not receive TPT (21/22, 95% vs. 122/312, 39%, p < 0.001). We did not observe any significant differences in TPT coverage by sex and urban/rural residence. Compared to children from nuclear families, a significantly higher proportion of children from extended families did not receive TPT(27/46, 59% vs. 57/152, 37%, p = 0.038). Children with a non-parent index case were less likely to receive TPT (23%) compared to those with a parent index case (77%, p = 0.008). (Supplementary Data Table S1)

Of the 143 children who were potentially eligible but did not receive TPT, 72 (50%) missed TPT due to provider-related reasons and 71 (50%) due to family-related reasons. The top provider-related reason reported was that they did not inform the family that the child needed a TPT assessment (n=51, 71%). Other provider-related reasons included that the children were exposed to drug-resistant TB (n=8, 11%) for which TPT formulations were not available or non-indication for TPT because the child was only exposed to EPTB (n=6, 8%). The top family-related reason was that the family declined the prescribed TPT (n=46, 65%). Other family-related reasons reported included that the family did not think TPT was necessary because the child was not living with the TB survivor after diagnosis and during the treatment (n=12, 17%) or because the child was born during the treatment (n=6, 8%) although the child was exposed to TB (see Figure).

**Figure.**
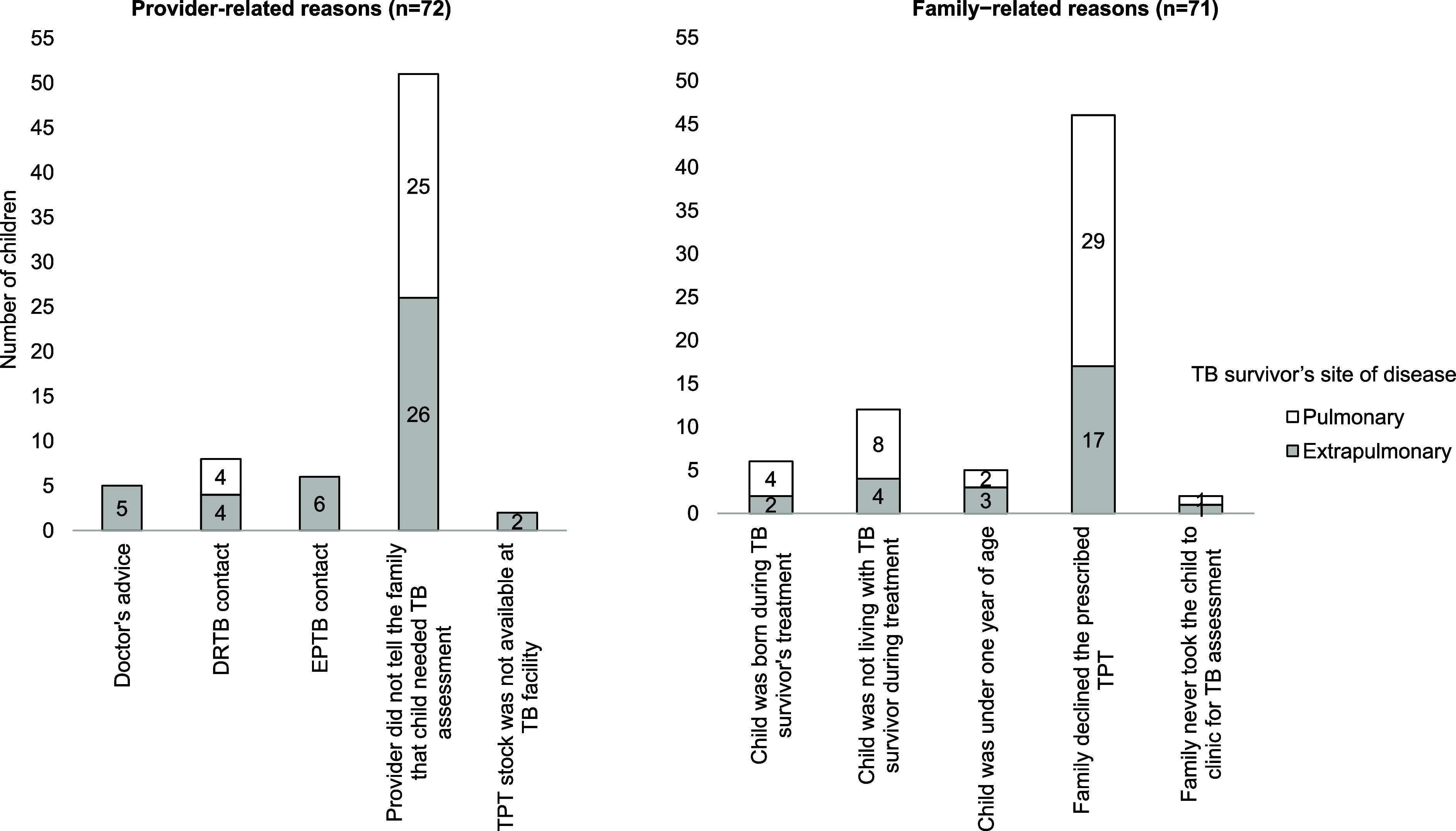
Reasons for not initiating TB preventive therapy (TPT) among child contacts less than 6 years of age during TB survivor’s recent TB treatment, stratified by TB survivor’s site of disease (n=143).

Among the 143 children who were potentially eligible for TPT in the post-treatment period, 128 (89%) completed 18 months of follow-up as of December 31, 2024. Among these, one was started on TPT, and two developed TBD. Of the remaining 125 children, 16 (13%) were re-exposed to TB in the post-treatment period. Reasons for not initiating TPT were documented at an 18-month visit for 94%(118/125) children. Despite our referrals for these eligible children during the post-treatment period, their TPT coverage remained unchanged. (Supplementary figure S1&S2) Though the overall provider-related reasons dropped from 51% (60/118) to 31% (37/118) at follow-up, the top provider-related reason (providers not informing families about the need for TPT) dropped from 34% (40/118) to 13% (15/118) and the top family-related reason (families opting out of TPT) showed a slight reduction from 34% (40/118) to 31% (36/118). The family-related reason (the family never took the child to the clinic for TPT assessment) increased from 1% (1/118) to 25% (29/118), see Supplementary Data Figure S2.

The observed TPT coverage of 67% among child contacts exposed to microbiologically-confirmed PTB in our study aligns with coverage for Maharashtra state more broadly.^10^ While nationwide TPT coverage in this group has been rising in recent years, it has not exceeded 60%.^10^ Though we observed no significant differences in coverage by urban/rural residence and sex, these factors as well as family type, have been identified as barriers by earlier studies.^13,14^ Children not living in nuclear families and those with a non-parent index case were less likely to receive TPT compared to their counterparts. In a prior study from India, these two factors were also identified as barriers to coverage related to lack of appropriate TPT knowledge and disclosure stigma.^13^ Younger children (<1 year) were identified as an additional barrier by this study. At treatment initiation, two key reasons for missed TPT initiation in our study were: (1) providers not informing families about the need for TPT and (2) families opting out of TPT. Though the prior reason reduced drastically, the latter did not change much during the 18-month visit. TPT coverage remained poor when reassessed at the 18-month visit mainly because families opted out and were reluctant to take the child to the clinic for TPT assessment. We recommend future qualitative studies to explore and understand provider and family-level factors for effective shared decision-making. Additionally, to improve TPT coverage among child contacts in India, we recommend strategies that support healthcare workers in generating demand among affected families. These may include enhanced trainings for TB providers on effectively counseling parents during and after treatment about the safety and efficacy of TPT, especially among children under one-year-old and from joint/extended families.

## Supplementary Material


