# Supplementary material for: TB preventive treatment coverage among children under six years of age in TB-affected households
Source: IJTLD Open. 2025 Nov 12;2(11):695–7. doi: 10.5588/ijtldopen.25.0207 (PMC12617088; doi:10.5588/ijtldopen.25.0207)
Supplement: Supplementary file 1 [file ijtldopen25-0207_supplementarydata1.pdf]

SUPPLEMENTARY DATA:

Supplementary table S1: Characteristics of children stratified by their TB preventive therapy uptake at TB survivor's treatment completion.

Characteristic	Child received TPT n (%)[a] n=191	Child didn't receive TPT n (%)[a] n=143	Overall n (%)[a] n=334	p-value[b]
**Age of the child at TB survivor's treatment completion, year (median, IQR)[c]**	3.00 (2.00, 5.00)	3.00 (1.50, 5.00)	3.00 (2.00, 5.00)	0.089
**Age category of the child at TB survivor's treatment completion, year[c]**				**<0.001**
<1 year	1 (1)	21 (15)	22 (7)	
1 to <6 year	169 (88)	111 (78)	280 (84)	
6 years and above	21 (11)	11 (8)	32 (10)	
**Gender**				0.9
Male	97 (51)	74 (52)	171 (51)	
Female	94 (49)	69 (48)	163 (49)	
**Family Type**				**0.038**
Nuclear	95 (50)	57 (40)	152 (46)	
Joint	77 (40)	59 (41)	136 (41)	
Extended	19 (10)	27 (19)	46 (14)	
**Residence**				0.2
Urban	129 (68)	86 (60)	215 (64)	
Rural	62 (32)	57 (40)	119 (36)	
**TB survivor's relation to the child**				**0.008**
Parent	148 (77)	92 (64)	240 (72)	
Non-parent	43 (23)	51 (36)	94 (28)	
**TB survivor's site of the disease in recent episode**				**<0.001**
Pulmonary involvement	148 (77)	73 (51)	221 (66)	
Extrapulmonary only	43 (23)	70 (49)	113 (34)	
**TB survivor's drug susceptibility in recent episode**				0.10
Drug-sensitive TB	186 (97)	133 (93)	319 (96)	
Isoniazid-resistant TB	2 (1)	6 (4)	8 (2)	
Multidrug-resistant TB	3 (2)	4 (3)	7 (2)	

a. Column-wise proportions are provided, with percentages rounded.
b. Wilcoxon rank sum test for continuous variables; Pearson's Chi-squared test or Fisher's exact test for categorical variables.
c. The maximum age of the children by TB survivor treatment completion was seven. All these children (n=334) were reported to be under six by TB survivors when they started their recent TB treatment.

Abbreviations: TB=tuberculosis; TPT=TB preventive therapy; IQR=interquartile range.

Supplementary figure S1: TB preventive therapy uptake of children under six and their follow-up status in TB Aftermath study as of 31 December 2024.

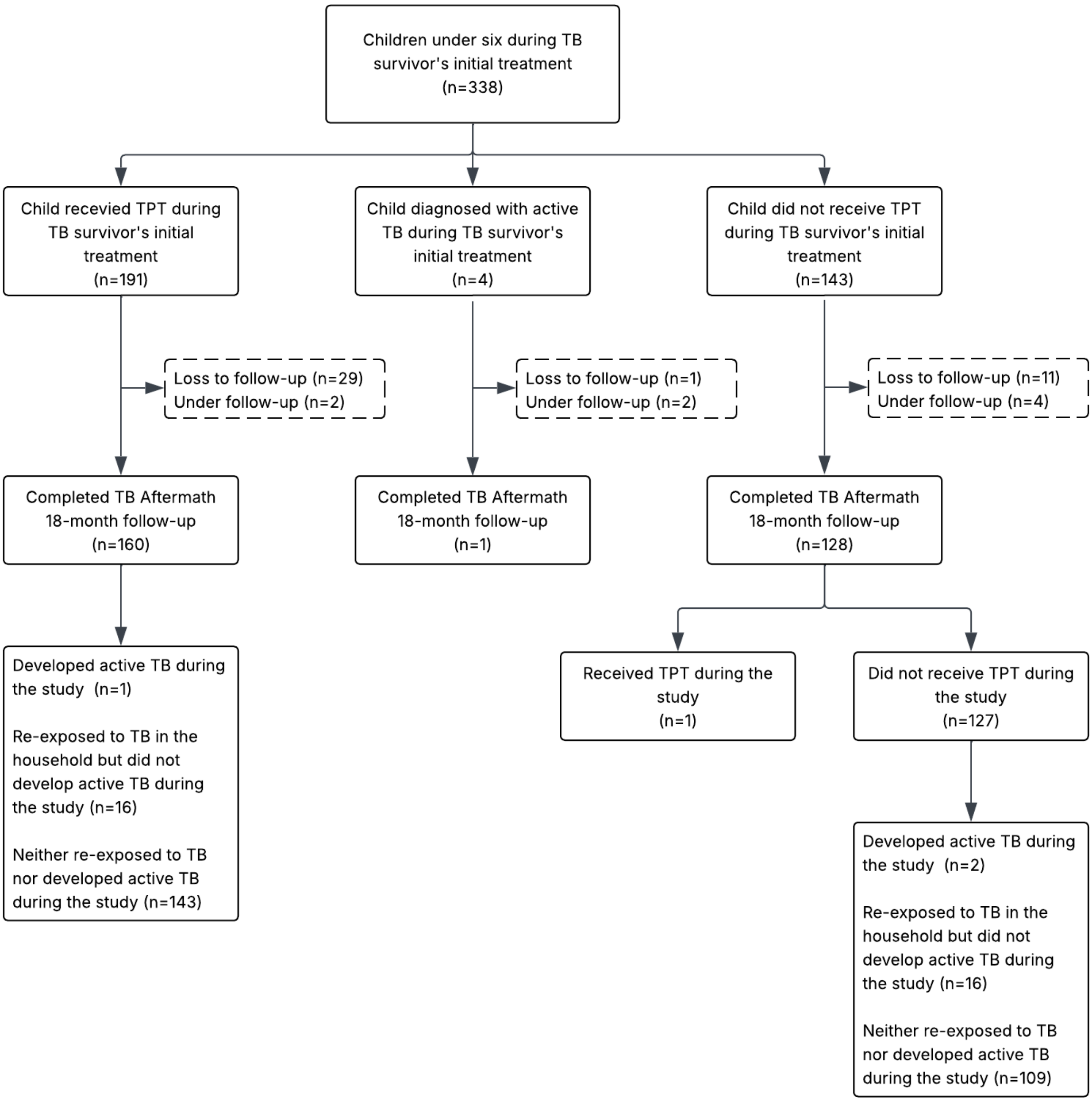

Abbreviations: TB=tuberculosis; TPT=TB preventive therapy.

Supplementary Figure S2: Reasons for not initiating TB preventive therapy in children during TB survivor's enrollment at TB Aftermath study and Month 18 study visit to the households (n=118).

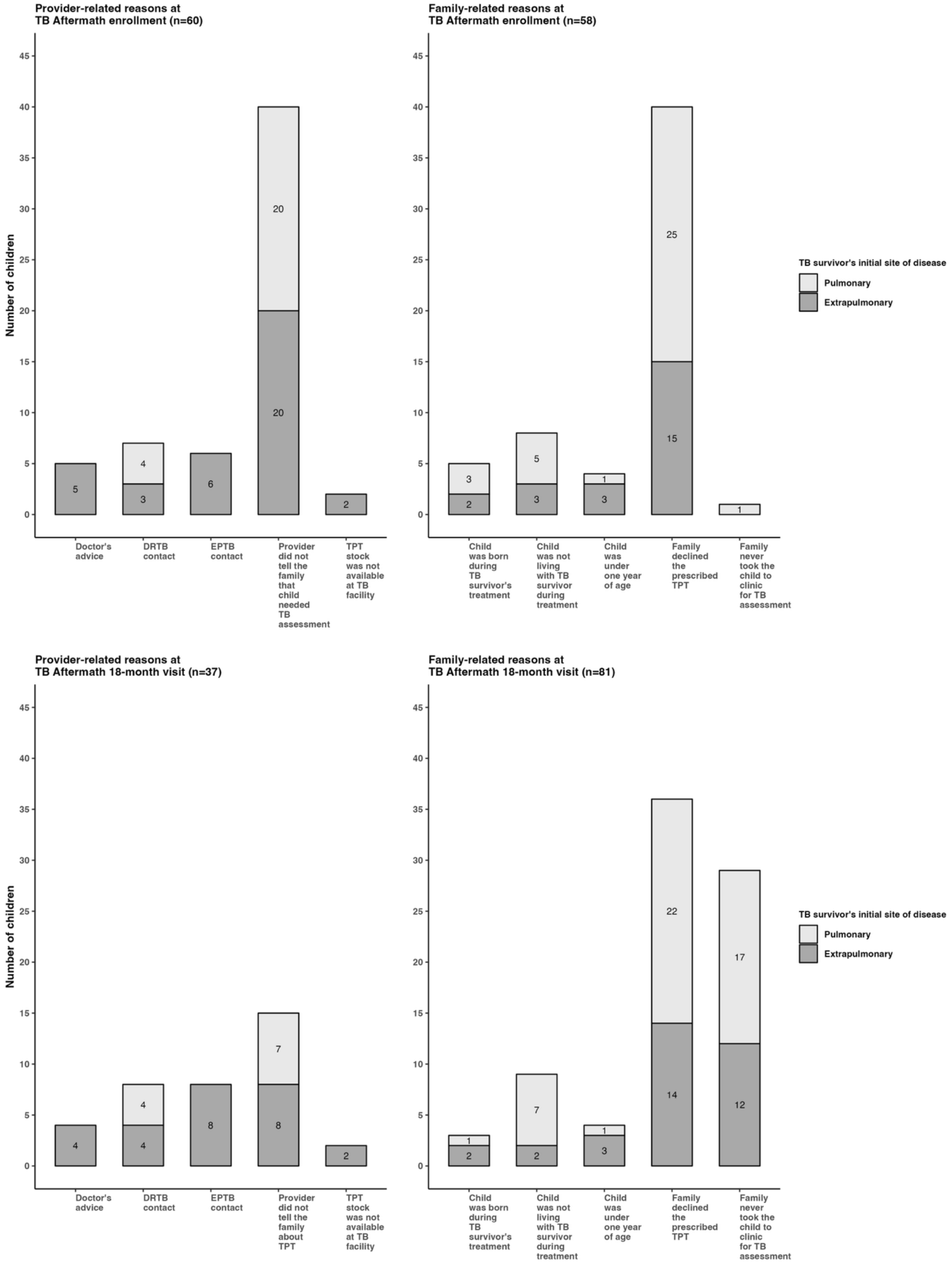

Among the 143 children who were potentially eligible for TPT in the post-treatment period, 128 (90%) completed 18 months of follow-up as of December 31, 2024. Of these, one child (1%) was initiated on TPT, and two (2%) developed TB disease. Among the remaining 125 children, 16 (13%) were re-exposed to TB during the post-treatment period. Reasons for not initiating TPT were documented for 118 of the 125 children (94%), based on information collected at enrollment in the TB Aftermath study and during the Month 18 household visits.

Abbreviations: TB=tuberculosis; TPT=TB preventive therapy; EPTB=extrapulmonary tuberculosis; DRTB=Drug-resitant tuberculosis.
